# Quality of care during childbirth at public health facilities in Bangladesh: a cross-sectional study using WHO/UNICEF ‘Every Mother Every Newborn (EMEN)’ standards

**DOI:** 10.1136/bmjoq-2018-000596

**Published:** 2019-08-24

**Authors:** Sk Masum Billah, Mohiuddin Ahsanul Kabir Chowdhury, Abdullah Nurus Salam Khan, Farhana Karim, Aniqa Hassan, Nabila Zaka, Shams El Arifeen, Alexander Manu

**Affiliations:** 1 Maternal and Child Health Division, International Centre for Diarrhoeal Disease Research Bangladesh, Dhaka, Bangladesh; 2 Epidemiology, University of South Carolina, Columbia, South Carolina, USA; 3 Health Promotion, Education and Behavior, University of South Carolina, Columbia, South Carolina, USA; 4 Health Section, Maternal and Newborn Health team, UNICEF Headquarter, New York City, New York, USA; 5 Department of Population Health, Liverpool School of Tropical Medicine, Liverpool, UK

**Keywords:** healthcare quality improvement, women's health, quality improvement, quality improvement methodologies

## Abstract

**Background:**

This manuscript presents findings from a baseline assessment of health facilities in Bangladesh prior to the implementation of the ‘Every Mother Every Newborn Quality Improvement’ initiative.

**Methodology:**

A cross-sectional survey was conducted between June and August 2016 in 15 government health facilities. Structural readiness was assessed by observing the physical environment, the availability of essential drugs and equipment, and the functionality of the referral system. Structured interviews were conducted with care providers and facility managers on human resource availability and training in the maternal and newborn care. Observation of births, reviews of patient records and exit interviews with women who were discharged from the selected health facilities were used to assess the provision and experience of care.

**Results:**

Only six (40%) facilities assessed had designated maternity wards and 11 had newborn care corners. There were stock-outs of emergency drugs including magnesium sulfate and oxytocin in nearly all facilities. Two-thirds of the positions for medical officers was vacant in district hospitals and half of the positions for nurses was vacant in subdistrict facilities. Only 60 (45%) healthcare providers interviewed received training on newborn complication management. No health facility used partograph for labour monitoring. Blood pressure was not measured in half (48%) and urine protein in 99% of pregnant women. Only 27% of babies were placed skin to skin with their mothers. Most mothers (97%) said that they were satisfied with the care received, however, only 46% intended on returning to the same facility for future deliveries.

**Conclusions:**

Systematic implementation of quality standards to mitigate these gaps in service readiness, provision and experience of care is the next step to accelerate the country’s progress in reducing the maternal and neonatal deaths.

## Background

In the last two decades, great strides have been made globally in reducing the maternal mortality ratio (MMR) and the under-five mortality rate (U-5MR), and both have fallen by over 44% and 56%, respectively.^1^ However, Millennium Development Goal (MDG) 5, of reducing the MMR by 75% its level in 1990 by 2015, was not met.^1^ In many countries, the fourth MDG on U-5MR was not achieved. Neonatal deaths decreased at a much slower rate than postneonatal deaths,^2^ resulting in an increased contribution of neonatal deaths to the overall U-5MR.^3^ In Bangladesh, neonatal death consists of nearly 60% of the overall U-5MR, although the country achieved the MDG target.^4^ The country also could not reach the target for MDG 5.^1^ This lag in neonatal mortality rate reduction and failure to meet MDG 5 are partly attributable to the quality of care for mothers and babies around the time of birth.^5^ At the current rates of reduction, Bangladesh is unlikely to achieve the Sustainable Development Goal (SDG) targets unless the quality of essential interventions for mothers and newborns are improved.^6^

Mothers and newborns, though separate entities, are closely linked and should not be viewed in isolation when combating the maternal and neonatal mortality.^7^ The first 24 hours after delivery are crucial to both mothers and newborns^3 8 9^ as 25%–45% of all neonatal deaths^8^ and the majority of maternal deaths occur during this period.^10 11^ Although facility deliveries have increased in the last decade, evidence suggests that higher levels of facility delivery do not necessarily translate into increased survival of mothers and newborns unless coupled with improved quality.^12–14^ Ensuring quality care around the time of birth and the immediate postpartum period can result in triple return aversion of maternal, fetal (stillbirth) and newborn deaths.^15^

The significance of quality of care around the time of birth has shaped many policies and initiatives such as the Every Newborn Action Plan (ENAP) and Ending Preventable Maternal Mortality (EPMM).^16 17^ Many of the strategic objectives of ENAP and EPMM revolve around improving the quality of care given to mothers and newborns.^16 17^ There is also a demand for a unified quality improvement (QI) model with a standardised model of service delivery, monitoring and accountability.^18 19^ To address this global imperative, the Every Mother Every Newborn QI (EMEN-QI) initiative was created.^20 21^ Ten EMEN ‘standards’ were developed to guide how health facilities provide evidence-based care to mothers and newborns with ‘criteria’ to measure these standards (online supplementary appendix S1).^22^ These standards are being tested in three countries—Ghana, Tanzania and Bangladesh—taking advantage of the ongoing Mother and Baby Friendly Hospital Initiative funded by the Bill and Melinda Gates Foundation and UNICEF.^21^ In Bangladesh, the initiative is being implemented jointly by the Government of Bangladesh (GoB), UNICEF headquarters and UNICEF Bangladesh.^21^

10.1136/bmjoq-2018-000596.supp1 Supplementary data

To assess the feasibility and the key requirements for strengthening facilities in readiness to implement EMEN standards, a baseline cross-sectional assessment was conducted to determine the status of quality of care in the selected government health facilities. This paper presents the findings from the baseline assessment in terms of structural readiness of facilities, availability of human resources, practices of healthcare providers and the experience of care as reported by clients. The findings have programmatic implications as they will inform on a process and tools to identify priority interventions for implementation and scale-up in similar low-income and middle-income countries (LMICs).

## Methodology

### Study design and setting

The EMEN-QI initiative adopts a quasi-experimental design with ‘intervention’ and ‘comparison’ districts that are being assessed before and after the implementation of the initiative. The baseline cross-sectional survey was conducted between June and August 2016 in 15 selected government health facilities of Kurigram, Lalmonirhat and Gaibandha districts of Rangpur division in Northwestern Bangladesh. From each of the districts, the district hospital (DH) and four subdistrict health facilities (Upazila Health Complexes, UHCs) were chosen. All three DHs offer comprehensive emergency obstetric care (CEmOC) services and have between 100 and 250 beds. Conversely, all UHCs were 31–50 bedded facilities, designated to provide at least basic emergency obstetric care; and three of them provided CEmOC services.^23^

### Framework for assessing the quality of care around the time of birth

The quality of maternal and newborn care in health facilities was assessed using the Donabedian’s three-dimensional conceptual framework for assessing the quality of care which involves structure, process and outcome.^24^ The key areas assessed under each of these domains are outlined in table 1 and elaborated below.

**Table 1:** Domains, key areas, data collection methods and data sources for assessing the quality of care

Domain	Key areas assessed	Data collection method	Data source or respondent
Structure	Physical environment and basic amenities Availability of essential drugs and equipment Readiness of referral system	Structured observation of health facilities using a checklist	15 health facilities 3 DH 12 subdistrict hospitals or UHC
	Human resources and training status	Document review, human resource record and interview of healthcare providers with a structured questionnaire	134 healthcare providers providing maternal and neonatal services 39 doctors 95 nurses
Process	Services provided during labour, delivery and immediate newborn care	Observation of labour, normal delivery and immediate newborn care	Observation of cases: Assessment at admission: 317 1st stage of labour: 272 3rd stage of labour: 297 Immediate newborn care: 287
	Documentation of health information	Document review	Delivery record review: 2323 including 150 caesarean section delivery
	Mechanism available in the facility to ensure patient rights and respectful care	Interview with a structured questionnaire	15 health facility managers
Outcome	Women’s perception of care received and their satisfaction with its quality	Interview with a structured questionnaire	295 recently delivered women at health facilities

DH, district hospital; UHC, Upazilla health complex.

### Structure: preparedness of facilities

As part of structural assessment physical environment and basic amenities such as water, sanitation and hygiene facilities available in the maternity wards of the assessment facilities were observed using a Bangladesh adapted version of a checklist predeveloped for this assessment in three countries. In addition, the availability of special care newborn unit (SCANU) in DHs and neonatal stabilisation units (NSU) in UHC, essential drugs and the functionality of essential equipment were assessed. The availability of the human resources and training provided to staff in maternal and newborn care was also assessed. The information on training received was collected from 39 doctors and 95 nurses who were providing maternal and neonatal services on the assessment days in 15 facilities and provided consent.

### Process: provision of care

Evidence-based practices around childbirth and during immediate postpartum care were assessed by (1) observing the care provided to pregnant women at different stages during labour and normal delivery using a checklist and (2) a review of facility records of all deliveries (including caesarean sections) in the 6 months prior to the data collection. In total, 317 normal deliveries were observed during the data collection period. Information extracted from facility records included the women’s background characteristics, antenatal care attendance, previous deliveries, obstetric history including the present pregnancy, labour and complication management, type of delivery, delivery outcomes and postnatal counselling. These data were extracted with a structured form developed together by the three countries, GoB’s delivery registers and record keeping forms. These records were categorised, by the frequency of maintenance, as ‘often recorded’ (recorded in >80% of forms), ‘sometimes recorded’ (10%–80%) and ‘rarely recorded’ (recorded in <10% of forms).

When mothers and newborns cannot be cared for at health facilities, they are referred to higher levels. We, therefore, assessed the availability of referral services in all facilities through questionnaire interviews with the health facility managers. Provisions within facilities to ensure the improved patient experience of care was also assessed through interviews with the managers. This covered the availability of complaints management systems and focal persons to receive client complaints; mechanisms of detecting patient abuse; and the presence of patient-support groups within these facilities.

### Outcome: client satisfaction with care

A total of 295 women, who delivered in all 15 assessment facilities, were interviewed using a structured questionnaire, just before they left for home after discharge, to obtain their perceptions on the experience of care during their stay in the facility. They were asked questions around privacy during care, their duration of stay in health facilities, care provider attention given to their newborns, their attitude and responsiveness of the healthcare providers, the opportunity to ask questions, the counselling they received on breastfeeding and postnatal care, and whether they would like to revisit the facility or recommend it to others seeking similar services.

### Organisation of fieldwork

#### Data collector recruitment

The data collectors were locally recruited to ensure that they were better adapted to the distinct culture of the northern parts of Bangladesh and would speak the local languages where required. They included research physicians and research assistants with social science backgrounds who led specific aspects of the data collection based on their expertise. The research physicians observed childbirths and interviewed health managers and healthcare providers. The research assistants conducted the assessment of the health facility structures and the exit interviews with recently delivered women.

#### Data collection

Data collectors were trained on the data collection instruments at a 7-day workshop facilitated by assessment investigators and a consultant from UNICEF who ensured uniformity in the assessment approach across the three countries. All the standardised data collection instruments were translated into Bangla (the local language), pretested and contextually adapted to fit the Bangladesh-specific culture and practices. Data were collected by three separate teams in the three districts; each team comprising two research physicians and two research assistants. Research physicians did not interact with patients or healthcare providers during observation of childbirth. The data collection teams also resided in the health facility premises for 2 weeks to ensure round the clock observation of giving childbirth. Two weeks stay in each facility allowed the data collectors to observe an estimated 15–20 childbirths per facility (determined based on facility-specific monthly delivery load) and to capture practice during weekends.

The study investigators conducted quality assurance around data collection through supportive supervision in the form of biweekly field visits to physically verify the data on the modules, review a sample of case observation forms for internal consistency and completeness, and cross-check these with the source documents, if available, for accuracy. One refresher training session was done for the data collectors after few days of the onset of data collection to resolve the problems that arose in the initial phase of data collection.

### Data processing and analysis

#### Data management

Data were collected on paper-based forms and were checked by the research physicians for completeness and consistency of coded responses before submitting to the study management team. Problem forms were sent back to the field for verification and correction. If inconsistency was found in time-variant data, health facility records were checked if available, otherwise, the data were declared as a missing value. Cleaned data were stored on a password-protected dedicated server. Only the study management team had access to the data.

### Statistical analysis

All data were analysed using Stata V.13 (StataCorp). Data were represented using graphs, tables and numerical measures. Simple tabulations and cross-tabulations of key outcomes were done. Z-tests were used to test differences in proportions.

### Patient and public involvement

The research questions and outcome measures were related to assessing the quality of maternal and neonatal care in the selected government hospitals. Pregnant women in labour, their newborns and family members were involved in the study as they were observed while receiving care in the health facilities and interviewed to explore their satisfaction with the health services. Patients or pregnant women were not involved in the design of, in the recruitment to and conduct of the study. The results will be disseminated among the health managers and health workers in the government hospitals where the study was conducted and will be reflected in the quality of patient–provider interactions and improving the patient’s right in those health facilities.

Written informed consent was sought from the study participants, including the healthcare providers and the women in labour before any observation or interview began. A predeveloped study information sheet explaining study purpose and participant’s rights was read out to the participant.

## Results

### Structural preparedness

#### Physical environment

All 15 health facilities (3 DHs and 12 UHCs) were assessed in this baseline study. As shown in table 2, only one DH and four UHCs had a separate maternity ward. All health facilities had labour room, but one in every three did not have a screen or curtain to ensure the visual privacy of clients in labour. Twelve of the facilities had separate toilets for pregnant and recently delivered women, but none of the toilets was adjudged clean by the data collectors. No facility had puncture-proof containers for sharps disposal. GoB protocols for infection prevention and control were not available in any of the 15 health facilities assessed.

**Table 2:** Structural preparedness at the selected health facilities

Indicators	Overall (n=15)	DH (n=3)	UHC (n=12)
Physical environment and basic amenities			
Separate maternity ward	6	2	4
Separate labour room	15	3	12
Screen or curtain for privacy in labour room	10	3	7
Separate toilet for pregnant and recently delivered woman	12	2	10
Toilet with hand washing facility	7	2	5
Clean toilet	0	0	0
Waste bins	10	3	7
Colour coded waste bins	1	0	1
Puncture proof containers at facility	0	0	0
Government protocol for infection prevention and control	0	0	0
Display chart with numbers of deliveries and sick newborns	15	3	12
Specialised care for newborn			
Separate neonatal corner	11	3	8
Availability of specialised unit*	2	2	0
Radiant warmers	2	0	2
Availability of KMC ward/beds	1	0	1
Essential drugs that are always available			
Intravenous fluid (normal saline or ringer’s lactate)	15	3	12
Inj. magnesium sulfate	2	2	0
Inj. diazepam	7	3	4
Inj. oxytocin	*	*	0
Inj. steroid	7	3	4
Inj. antibiotic (Ampicillin/Penicillin/Gentamicin/Cephalosporin)	4	3	*
Equipment that are functional			
Wall clock with a secondhand	0	0	0
Freezer/fridge for storing medicine	12	2	10
Glucometer	15	3	12
Pulse oximeter	3	2	1
Bag and mask for newborn	15	3	12
Penguin sucker	10	3	7
Filled oxygen cylinder	15	3	12
Paediatric nebulizer	1	0	1
Readiness of the referral system			
Availability of referral system for mother and newborn	14	2	12
Ambulance available round the clock	13	2	11
Patients have to pay for using the ambulance	10	2	8
Referral facilities contacted over phone before sending patients	7	1	6

*Availability of SCANU was assessed in DH and NSU in UHC as specialised care unit.

DH, district hospital;KMC, kangaroo mother care; NSU, neonatal stabilisation unit; SCANU, special care newborn unit; UHC, Upazilla health complex.

#### Specialised services

Table 2 shows that all three DHs had separate newborn care corner in the labour room where babies were provided immediate newborn care. Only 8 (66.7%) UHCs had such corner. Only one facility was equipped to provide kangaroo mother care (KMC) for low birthweight and premature babies. No UHC had NSU for newborns while two out three DHs had SCANU to provide specialised care for newborn.

#### Essential drugs

Intravenous fluids such as normal saline or Ringer’s lactate were available across all 15 facilities. Only two DHs never had stock-outs of magnesium sulfate (MgSO4) injections, for the management of severe pre-eclampsia; but there were reported stock-out days in almost all the UHCs. Similarly, there were stock-outs for oxytocin, a uterotonic, in all but 1 of the 15 facilities visited. Three of the facilities did not have refrigerators or freezers for the storage of heat-sensitive medicines or products.

#### Key equipment

Key equipment for managing maternal and newborn complications were not available or functional in many of the facilities assessed (table 2). Although all 15 facilities assessed had a bag and a mask for neonatal resuscitation, only two DHs and one UHC had pulse oximeter, a critical equipment in the management of newborn complications. Five facilities did not have a penguin sucker for clearing the airways of newborns during resuscitation.

#### Referral services

Thirteen facilities had ambulances available round-the-clock to transfer the referred clients. However, less than half of these (7 out of 15) had provisions in their referral protocols to contact the referral destination facility before referring any patient. In two out of every three health facilities, referral services attracted out-of-pocket payment from patients for use of the ambulance.

#### Human resources

A shortage of health workforce, particularly obstetricians and paediatricians, was a predominant finding in most of the UHCs. Table 3 shows that only 17% of obstetrician and 8% of paediatrician sanctioned posts were filled at the time of the assessment. In addition, medical officer positions were mostly vacant at DHs. Anaesthetists were also lacking in many facilities with only 33% posts filled at DH and 25% in UHCs. Overall, 68% posts for nurses were filled, however, greater gaps were found at the subdistrict level compared with the DHs (53% vs 84% filled posts, respectively).

**Table 3:** Status of human resources in selected health facilities

Post	DH		UHC	
	No of sanctioned post	No of filled post	No of sanctioned post	No of filled post
Obstetrician	5	4	12	2
Paediatrician	6	4	12	1
Anaesthetist	6	2	12	3
Medical officer	43	13	82	54
Nurse	142	119	144	76

DH, district hospital; UHC, Upazilla health complex.

#### Training status of healthcare providers in care during pregnancy and for newborns

The study found that nurses had significantly higher training coverage than the doctors on care of pregnant women (99% vs 41%; p<0.001) and management of pregnancy complications (96% vs 41%; p<0.001). Overall, 44.8% of all doctors and nurses were trained in the management of newborn complications. Although there were slightly higher proportions of doctors than nurses with this training (51.3% vs 42.1%), the difference was not statistically significant.

### Process: provision of care

#### Initial care at admission with labour

Our results show that, at baseline, for the 317 women who presented in labour, many of the vital checks including blood pressure, pulse, fetal heart rate, temperature and urine assessment were not done (table 4). On arrival at health facilities with labour pain, 52% had their blood pressure measured; 22% had their pulse measured and only 1 in 10 were checked for pedal oedema. Urine was not assessed for proteins in almost all the pregnant women except for three (1%). Checks to assess the health and well-being of fetus were largely ignored; only in 15% of cases fetal heart rate was assessed.

**Table 4:** Evidence-based care as observed around the time of birth

Activities	Overall	DH	UHC	P value
Assessment at admission	**n=** **317**	**n=** **136**	**n=** **181**	
Blood pressure monitored	52.4	59.6	47.0	0.064
Pulse counted	22.1	10.3	30.9	<0.001
Abdomen examined	48.6	47.1	49.7	0.472
Fetal heart rate monitored	14.8	6.6	21.0	<0.001
Temperature measured	1.9	2.2	1.7	0.723
Urine assessed	1.0	0.7	1.1	0.737
Per-vaginal examination conducted	93.1	90.4	95.0	0.272
All of the above examinations received	0	0	0	–
During the first stage of labour	**n=** **272**	**n=** **111**	**n=** **161**	
Started partograph for monitoring labour	0	0	0	–
Women examined for vitals in labour ward	91.5	89.2	93.2	0.060
During the third stage of labour	**n=** **297**	**n=** **141**	**n=** **156**	
Oxytocin given as part of AMTSL	82.5	94.3	71.8	<0.001
Uterine massage immediately after delivery	60.3	58.9	61.5	0.491
Care of newborns for all live births	**n=** **287**	**n=** **132**	**n=** **155**	
Spontaneous breathing assessed	90.2	93.2	87.7	0.122
Dried immediately and thoroughly	97.6	98.5	96.8	0.349
Put on skin to skin immediately after birth	27.2	24.2	29.7	0.198
Delayed cord clamping (after 1 min)	69.3	75.0	64.5	0.210
Cutting cord with sterile blade	61.0	70.5	52.9	0.002
Breastfeeding initiated within 1 hour	62.0	76.5	49.7	<0.001
Chlorhexidine applied to umbilical cord	57.1	61.2	53.6	0.409
Birth weight taken	75.6	86.4	66.5	<0.001
All seven components of immediate newborn care* practiced	17.4	18.2	16.8	0.979
Management of birth asphyxia†	**n=** **77**	**n=** **30**	**n=** **47**	
External stimulation or resuscitation given	97.4	96.7	97.9	0.746

*Immediate newborn care practices: assessment of spontaneous breathing, drying immediately and thoroughly, skin-to-skin contact, delayed cord cutting, cutting cord with sterile blade, initiation of breast feeding within 1 hour and application of chlorhexidine to umbilical cord.

†Newborn who did not cry or breathe spontaneously at birth.

AMTSL, active management of the third stage of labour; DH, district hospital; UHC, Upazilla health complex.

#### Care during labour

Although the majority (93%) of the women received a vaginal examination at initial assessment to confirm the stage of labour, labour management, thereafter, was often poor. Partographs were not used at all to monitor labour in all the facilities. Once the baby was delivered, 17% of women did not receive oxytocin as part of the management of the third stage of labour. DHs were significantly more likely to administer oxytocin after birth than UHCs (94% vs 72%; p<0.001).

#### Care of the newborn after delivery

Seventeen per cent of newborns were provided care covering all seven components of immediate newborn care and practices did not significantly differ between DHs and UHCs (p=0.697). Almost all babies were dried immediately after birth but only 27% were put in skin to skin with the mother’s abdomen. Breast feeding was initiated within 1 hour in 77% of mothers in DHs as compared with 50% in UHCs (p<0.001). Chlorhexidine was applied to the umbilical cord only in 57% of all live births. Beyond the stimulation from drying, it is important to note that nearly 97% of the newborns who did not cry spontaneously received external stimulation or resuscitation.

#### Documentation of health information

A total of 2323 client records covering all deliveries (including 150 caesarean sections) conducted within 6 months of the survey were reviewed to assess the quality of the documentation process and content of care. It was identified that the mother’s age, gravidity, parity, type of delivery and the newborn’s health outcomes were ‘often recorded’. However, other important patient information that has a direct bearing on subsequent care, such as previous history of abortion, stillbirths, caesarean sections and the presence of chronic diseases, were ‘rarely recorded’. No record form was found with an accompanying partograph. Among the records of caesarean sections, surgical notes along with a rationale for performing caesarean sections were rarely found (table 5).

**Table 5:** Status of recorded information in the delivery registers and record keeping forms

Often recorded (>80%)	Sometimes recorded (10%–80%)	Rarely recorded (<10%)
Record forms of all deliveries including caesarean section, n=2323		
Mother’s age No of conceptions (gravida) No of live births (parity) Type of delivery Health outcome of newborn Birth weight	Mother’s gestational age Antenatal visit status Status of membrane Status of presentation (normal or malpresentation) Pre-eclampsia or eclampsia Health outcome of mother	Mother’s education and occupation History of abortion, stillbirth or caesarean section History of any chronic disease Predischarge summary Partograph*
Record forms of caesarean section deliveries, n=150		
Name of the surgeon Type of anaesthesia	Postcaesarean antibiotics administered Postcaesarean fluid management specified	Surgery notes Indication for caesarean section Complications of surgery

*Never recorded.

#### Provisions for receiving information on client perceptions of care

All 3 DHs and 3 of the 12 UHCs had complaint/suggestion boxes. Six out of 15 facilities had focal persons to receive the client complaints about the care. In 60% of facilities, managers reported that a mechanism was in place to identify any kind of abuse of women during maternity care.

### Outcome or satisfaction of clients

**Figure 1:**
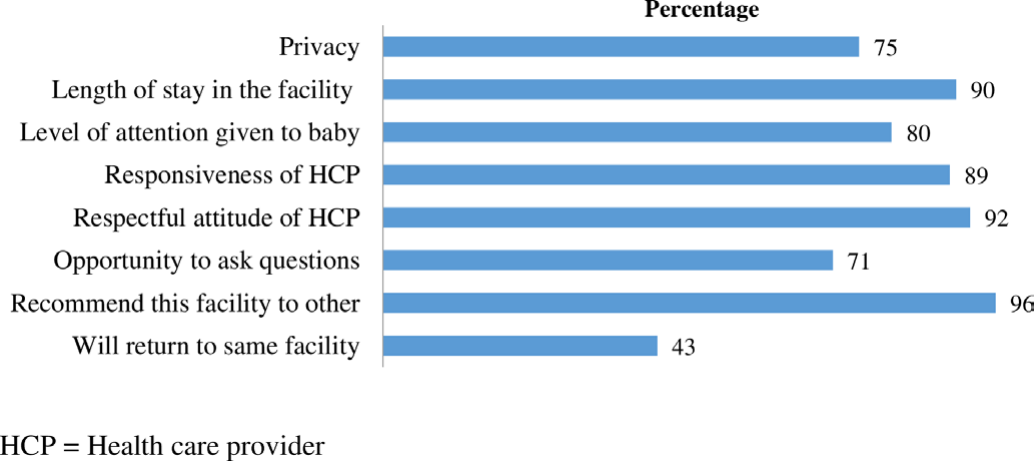
Women satisfied with the services received from health facilities (n=295).

A total of 295 delivered women were interviewed to assess their satisfaction with the maternity services received. Most of the women were satisfied with the privacy maintained, length of their stay, healthcare providers’ responsiveness and respectful attitude (figure 1). Although 96% of the women interviewed said they would recommend the health facility to others to deliver there, only 43% said they themselves like to return to the same health facility for maternity services.

## Discussion

This cross-sectional survey assessed the quality of maternal and newborn services in selected primary and secondary level health facilities of three Bangladeshi districts using the Donabedian framework and corresponding indicators developed by the EMEN-QI initiative. Noteworthy gaps and inconsistencies were found in all three domains of the Donabedian model of assessment. Furthermore, the effects of some inadequacies in one domain compounded on those of other domains, resulting in suboptimal care. More specifically structural elements greatly limited the completion of essential maternal and newborn care processes. The conflicting finding that many patients were reasonably satisfied with their care but simultaneously uninterested in returning to the facility for future deliveries implies that patient satisfaction outcomes alone do not accurately reflect the experience of care.

A critical structural gap was found in the human resource, which includes the shortage of service providers and their lack of capacity to deliver appropriate care. Vacancies in service provider positions, particularly clinicians, are common in the public sector of Bangladesh and other LMICs and were corroborated by the findings of this study.^25 26^ The absence of obstetricians, paediatricians and anaesthetists in health facilities along with untrained birth attendants amplified the challenges in managing maternal and neonatal complication during labour, birth and the immediate postpartum period.^27^ The possibility of task-shifting in the management of serious complications from physicians to nurses did not appear feasible, as there were also shortages in the nurse workforce. Only 41% of doctors were trained on caring for pregnant women and managing their delivery complications, effectively lowering the number of capable providers and compounding the effects of vacant posts.

The availability of necessary supplies and medications is also categorised under structural components of care. Supplies of medications for pre-eclampsia, severe eclampsia and postpartum haemorrhage (PPH) are particularly significant, as these disorders are major causes of maternal deaths within Bangladesh and globally.^1 28^ Management of eclampsia or severe pre-eclampsia, both hypertensive disorders, is initiated by administering MgSO4 to mothers and maintained through additional dosages or obstetric intervention if required.^29^ Only two DHs and no sub-DH/UHC had continuously available supplies of MgSO4. Only one facility had the guaranteed availability of oxytocin, the first drug of choice for PPH management. The gaps in emergency drug availability are consistent with other studies conducted in health facilities of LMICs,^30–32^ including Bangladesh.^33^ The data from this study suggest that the effects of these supply limitations did not necessarily translate to the process of active management of the third stage of labour, a crucial step of postdelivery care to prevent PPH, as 83% of the women received oxytocin as part of AMTSL. This inconsistency could be explained by clients purchasing and bringing the medications to the facility, which was also observed by one study conducted in India^34^; however, no inference can be made about whether all patients had equitable opportunities to use oxytocin as costs and proximity to pharmacies may be barriers for certain patients.

In LMICs, wherein the absence of sophisticated labour monitoring tools, processes of identifying complications early and properly documenting the complications are essential to ensuring timely referrals and selecting the mode of delivery.^35 36^ Abdominal examinations, fetal monitoring and vaginal examination at regular intervals can help identify complications in labour early.^19^ Though WHO assigns great importance to routinely measuring blood pressure and assessing urine to detect pre-eclampsia,^29^ blood pressure was only checked in half of the women during labour and urine samples were rarely checked for proteins. These practices are also included in the labour management guideline developed as part of the Bangladesh Maternal Health Strategy,^37 38^ however, the study findings show poor compliance of the birth attendants with the protocol. Use of WHO safe childbirth checklist, a low-cost, and scalable intervention, has shown significant improvement in nurse–midwives’ compliance with essential childbirth practices.^39 40^ To further the implementation of the checklist at scale as a job aid in labour room, we recommend that the feasibility of using the locally adapted and validated checklist can be tested within the scope of EMEN-QI initiative in the selected district and sub-DHs to ensure appropriate maintenance of the labour protocol by the birth attendants.

The study identified poor documentation of critical indicators including indications for caesarean section and complications during delivery which is consistent with the findings of several other studies.^36 41^ The partograph is an important early warning tool recommended by WHO to monitor the progress of events during labour.^42^ However, none of the assessed facilities used the partograph, consistent with previous studies showing minimal use of the tool.^33 43^ Ensuring a regular supply of partographs, providing adequate training and supervision to ensure its use is required to improve overall outcomes of labour and the general documentation of vital information around labour management and delivery.^36 44^

The gaps continued beyond labour and delivery and were also present in immediate newborn care. All seven components of immediate newborn care were practised in only 17.4% of live births across the selected facilities. The element of care observed least often was putting a newborn infant in skin-to-skin contact with their mother, also known as KMC. Preterm neonates put in KMC have reduced rates of mortality as well as promoted earlier initiation of breastfeeding.^45 46^ Based on these positive results, the ‘National Core Committee on Neonatal Health’ of Bangladesh decided to scale up KMC, as reflected in ‘A Promise Renewed’ in 2013, however, the results of this assessment confirm that policy has not been translated to action.^47^

Client satisfaction or dissatisfaction helps to identify the strengths or weaknesses of the services provided to them.^48^ There was an evident disconnect between women’s reported satisfaction with care received and the suboptimal physical environment of facilities. In several other studies, similar high levels of satisfaction were reported from the clients’ perspectives despite the provision of poor services.^49 50^ Possible reasons may be the clients’ lack of awareness regarding rights to dignified care services or fear of denial of health services in future.^51 52^ The intention to use the service from the same health facility again could be a better reflection of the client’s true impression of care.^53^ The fact that more than 93% of the clients would recommend the same facility to others whereas only 43% of the clients would return themselves, underscores the gaps in quality of care based on clients’ experiences. In the future, in-depth explorations of clients’ perspectives should be taken into account to develop patient-centred maternity care.^54^

While this study did not assess the impact of interventions on changing the structural, procedural or outcome-oriented elements of maternal and newborn care, it did identify where these deficiencies were. The challenge lies in selecting areas of care to prioritise, a necessity as Bangladesh has limited resources. Based on the findings from this study, the most critical gap in care was in human resources. This calls for the government to pay more attention to increasing the number of trained healthcare providers in primary and secondary level health facilities as well as providing avenues for continuous professional development; however, a system-level change of this calibre is not immediate. Therefore, in the interim, the capacity of services providers to deliver evidence-based care should be improved. As there are supply limitations, which again should be addressed through systematic changes, providers should maximise the coverage of practices such as the use of WHO safe childbirth checklist and partograph, and KMC practices that can be accomplished without additional human resources. This capacity gap can be overcome by regular training with supervisory support to translate knowledge to practice.^19 55^ Several systematic reviews suggest that regular audits, review of maternal and newborn complications and provision of feedback by clinical experts can substantially improve the quality of services delivered in health facilities.^56 57^ Coupling capacity building with regular monitoring may help providers to identify where improvements within their individual facilities are needed.

### Strengths and limitation

Our study assessed the quality of care around the time of birth through actual observations of normal labour cases from admission to discharge. Direct observation is considered the gold standard to evaluate any health programme or activity.^58^ The holistic approach to exploring the quality of care enabled the corroboration of information among facility managers, healthcare providers and clients, revealing gaps in maternity services that are generally difficult to explore. Although for some quality indicators the measurement relied on the manager’s interview only, for instance, availability of patient-centred care. Being aligned with WHO quality care framework,^59^ EMEN-QI initiative provides a model to assess and develop strategies to improve maternal and neonatal health through health system strengthening. Despite these strengths, this study also had limitations. Hawthorn effect was possible by the presence of observers. However, their continual presence over 2 weeks in the selected facilities could potentially minimise the Hawthorn effect. The study findings reflect on the quality of care around childbirth, however, the entire spectrum of EmOC services, including the management of complications in both mothers and newborns, was beyond the scope of the study.

## Conclusion

Quality of care in maternal and newborn service provision particularly at the time around childbirth has been recognised as a critical issue in the international (maternal and newborn) health agenda. This baseline assessment was a part of a greater series of assessments to evaluate whether the EMEN-QI initiative influenced the quality of care provided around the time of delivery. Using the EMEN standards, the study identifies gaps in structural readiness and provision of evidence-based maternity care in the health facilities providing obstetric care to both mothers and newborns. While increasing human resources and building the capacity of the current workforce are important, ensuring that supplies of life-saving drugs, such as MgSO4 and oxytocin, are available in all public facilities as well as essential components of newborn care and partograph usage should be prioritised in immediate implementation. Longer term strategies should be undertaken to retain the expert physicians and anaesthetists in rural and underserved areas building the facilities’ capacities and reducing the need for referrals. Addressing such critical barriers along with strengthening the capacity and competency of the maternity care providers can accomplish the goal of preventing maternal and neonatal mortality in countries with the similar challenges.
