# Supplementary material for: Quality of care during childbirth at public health facilities in Bangladesh: a cross-sectional study using WHO/UNICEF ‘Every Mother Every Newborn (EMEN)’ standards
Source: BMJ Open Qual. 2019 Aug 24;8(3):e000596. doi: 10.1136/bmjoq-2018-000596 (PMC6711449; doi:10.1136/bmjoq-2018-000596)
Supplement: Supplementary data [file bmjoq-2018-000596supp001.pdf]

Appendix S1: EMEN Standards

Clinical Care

1. Evidence-based safe antenatal care is provided.
2. Evidence-based safe care is provided during labor and childbirth.
3. Evidence-based safe postnatal care is provided for all mothers and the newborns.

Patients' Rights

4. Human rights are observed and the experience of care is dignified and respectful for every woman and newborn.

Crosscutting

5. A governance system is in place to support the provision of quality maternal and newborn care.
6. The physical environment of the health facility is safe for providing maternal and newborn care.
7. Qualified and competent staff is available in adequate numbers to provide safe, consistent and quality maternal and newborn care.
8. Essential drugs, supplies and functional equipment and diagnostic services are consistently available for maternal and newborn care.
9. Health information systems are in place to manage patient clinical records and service data.
10. Services are available to ensure continuity of care for all pregnant women, mothers and newborns.
